# Surveillance of seroepidemiology and morbidity of Chagas disease in the Negro River, Brazilian Amazon

**DOI:** 10.1590/0074-02760170169

**Published:** 2018-01

**Authors:** José Rodrigues Coura, Angela CV Junqueira, João Marcos BB Ferreira

**Affiliations:** 1 Fundação Oswaldo Cruz-Fiocruz, Instituto Oswaldo Cruz, Laboratório de Doenças Parasitárias, Rio de Janeiro, RJ, Brasil; 2 Universidade do Estado do Amazonas, Programa de Pós-Graduação em Medicina Tropical, Manaus, AM, Brasil; 3 Fundação de Medicina Tropical Dr Heitor Vieira Dourado, Manaus, AM, Brasil

**Keywords:** surveillance, seroepidemiology, Chagas disease, Rio Negro, Brazilian Amazon

## Abstract

**BACKGROUND:**

Chagas disease in the Brazilian Amazon Region was previously regarded as an enzootic disease of wild animals. More recently, in situations where humans have penetrated the wild ecotope or where triatomines and/or wild animals (marsupials) have invaded human homes resulting in disease transmission, Chagas disease has come to be regarded as an anthropozoonosis. We found that the highest incidence of infection due to *Trypanosoma cruzi* and Chagas disease occurred among piassaba fibre gatherers and their families.

**OBJECTIVES:**

Considering the results of previous surveys, we conducted a new survey of piassaba gatherers and their families in the creeks of the Aracá, Curuduri, Demini, Ererê and Padauiri rivers, which are tributaries on the left bank of the Negro River, in the municipality of Barcelos; Barcelos-Caurés highway; Negro River in Santa Isabel of the Negro River; and Marié River, on the right bank of the Negro River.

**METHODS:**

A questionnaire was applied to 482 piassaba gatherers and their families who accompanied them. We collected 5-mL blood samples (with permission from each subject), separated the serum, and performed serological tests using indirect immunofluorescence and conventional and recombinant enzyme-linked immunosorbent assays (ELISA). We performed brief clinical examination and electrocardiograms. Only 273 subjects attended our field base for detailed clinical examination and electrocardiogram.

**FINDINGS AND MAIN CONCLUSIONS:**

The questionnaire revealed that 100% of the 482 patients recognised the triatomine *Rhodnius brethesi*, which they had seen in the piassaba plantation and 81% in their field huts. A total of 79% of subjects had previously been bitten by this vector and 21% did not know. The 25 subjects seropositive for *T. cruzi* infection (5.2%) stated that they had been bitten more than 10 times by this insect. Of the 273 subjects who underwent electrocardiogram, 22% showed conditions that were possibly attributable to Chagas disease or other cardiovascular disease.

Chagas disease in the Brazilian Amazon Region was previously regarded as an enzootic disease of wild animals, from the time when Chagas (1924) confirmed that its cause was *Trypanosoma cruzi*, a parasite found by Abhen-Athar in the common squirrel monkey (*Saimiri sciureus*), in the state of Pará in 1922. Since then, a variety of mammal species (marsupials, bats, rodents, carnivores, edentates, and primates) native to the Amazon Region have been described as reservoirs of *T. cruzi* (Coura & Junqueira 2012). Table I shows the different species that form wild reservoirs for *T. cruzi* in the Brazilian Amazon Region and the references for the respective authors.

**TABLE I:** Mammals from Brazilian Amazon found to be infected with *Trypanosoma cruzi*

Order	Species	References
Marsupialia (Didelphiomorphia)	*Calluromys spp*	Lainson et al. (1979), Miles et al. (1981) Rodrigues and Mello (1942), Deane (1961), Deane (1964a,b),
	*Didelphis marsupialis*	Deane (1967) Lainson et al. (1979), Miles et al. (1981) Póvoa et al. (1984)
	*Marmosa cinerea*	Deane and Jansen (1939), Lainson et al. (1979), Póvoa et al. (1984)
	*Metachirus nudicaudatus*	Deane (1958), Lainson et al. (1979), Miles et al. (1981); Póvoa et al. (1984)
	*Monodelphis brevicaudata*	Miles et al. (1981), Póvoa et al. (1984)
	*Philander opossum*	Deane (1958), Lainson et al. (1979), Miles et al. (1981)
Chiroptera *(T. cruzi ou T. cruzi-like)*	*Carollia perspicillata*	Dias et al. (1942)
	*Choeroniscus minor*	Dias et al. (1942)
	*Glossophaga sorcina*	Dias et al. (1942)
	*Lonchophylla mordax*	Dias et al. (1942)
	*Mycronycteris megalotis*	Dias et al. (1942)
	*Molossus major*	Deane (1961), Deane (1964a)
	Molossus ater	Deane (1961)
	*Phyllostomus hastatus*	Deane (1961, 1964a), Deane (1967)
	*Plyllostomus alongatus*	Dias et al. (1942)
	*Noctilio labialis*	Dias et al. (1942)
	*Saccopterix bilineata*	Dias et al. (1942)
Rodentia	*Agouti paca*	Lainson et al. (1979)
	*Coendou spp*.	Lainson et al. (1979), Miles et al. (1981)
	*Dasyprocta spp*.	Deane (1960), Lainson et al. (1979), Póvoa et al. (1984)
	*Echymys chrysurus*	Miles et al. (1981)
	*Nectomys squamipes*	Deane (1960)
	*Oryzomys capito*	Lainson et al. (1979), Póvoa et al. (1984)
	*Proechimys guayannensis*	Deane (1961), Lainson et al. (1979)
	*Rattus rattus*	Miles et al. (1981), Póvoa et al. (1984)
	*Sciurus spp*.	Miles et al. (1981), Póvoa et al. (1984)
Edentada (Xenarthra)	*Cyclopes ditactylus*	Rodrigues and Mello (1942), Deane (1961, 1964a), Lainson et al. 1979, Póvoa et al. 1984)
	*Dasypus novemcinctus*	
	*Tamandua tetradactyla*	Rodrigues and Mello (1942)
Carnivora	*Nasua nasua*	Lainson et al. (1979), Póvoa et al. (1984)
	*Tayra Barbara*	Ferreira and Deane (1938), Rodrigues and Mello (1942), Deane (1961,1964b)
Primates	*Cabuella pigmea*	Deane and Damasceno (1961)
	*Sanguinus fasciollis weddelli*	Ziccardi et al. (2000)
	*Sanguinus imperator imperator*	Ziccardi et al. (2000)
	*Saguinus midas niger*	Ziccardi et al. (2000)
	*Saimiri sciureus*	Miles et al. (1981) Chagas (1924), Deane (1964b), Ziccardi and Lourenço-de-Oliveira (1997)
	*Saimiri ustus*	Ziccardi and Lourenço-de-Oliveira (1997)

Source: Coura and Junqueira (2012).

Although at least 16 species of wild triatomine exist in the Brazilian Amazon Region and 10 have been found to be infected with *T. cruzi* (Table II), no triatomines with adaptation to human homes have been detected in this region, except for *Triatoma rubrofasciata*, an uninfected species exclusively found in port areas.

**TABLE II:** Triatomines found in the Brazilian Amazon

*Belminus herreri*	*Rhodnius brethesi* (+)
*Cavernicola lenti*	*Rhodnius nasutus*
*Cavernicola pilosa*	*Rhodnius neglectus* (+)
*Eratyrus mucronatus* (+)	*Rhodnius paraensis* (+)
*Microtriatoma trinidadensis* (+)	*Rhodnius pictipes* (+)
*Panstrongylus geniculatus* (+)	*Rhodnius robustus* (+)
*Panstrongylus lignarius* (+)	*Triatoma maculata*
*Panstrongylus rufotuberculatus* (+)	*Triatoma rubrofasciata*

Valente et al. (1998) found *Panstrongylus geniculatus* in pigsties on the Island of Marajó and Luitgards-Moura et al. (2005) found *Triatoma maculata* in hen-houses in agricultural colonisation areas in Roraima, peridomestic areas, and occasionally homes, but without adaptation. However, these situations indicate that the vectors are present in close contact with humans, and thus there is a future risk of adaptation.

Transmission of Chagas disease in the Brazilian Amazon Region may occur incidentally when humans invade the wild ecotope, when vectors or marsupials invade human homes, or through the faeces and urine of triatomines or secretions from the scent glands of marsupials (Fig. 1). This occurs in the following situations: (a) contamination of raw or cooked foods (*açaí* and others); (b) as a work-related illness of piassaba fibre gatherers (Fig. 2) and members of their families (Fig. 3); and (c) occasionally when triatomines come into contact with people in their homes and/or in the surrounding area ins search of food sources because of lack of reservoirs in enzootic areas (Coura et al. 1994, Coura 2013).

**Fig. 1:**
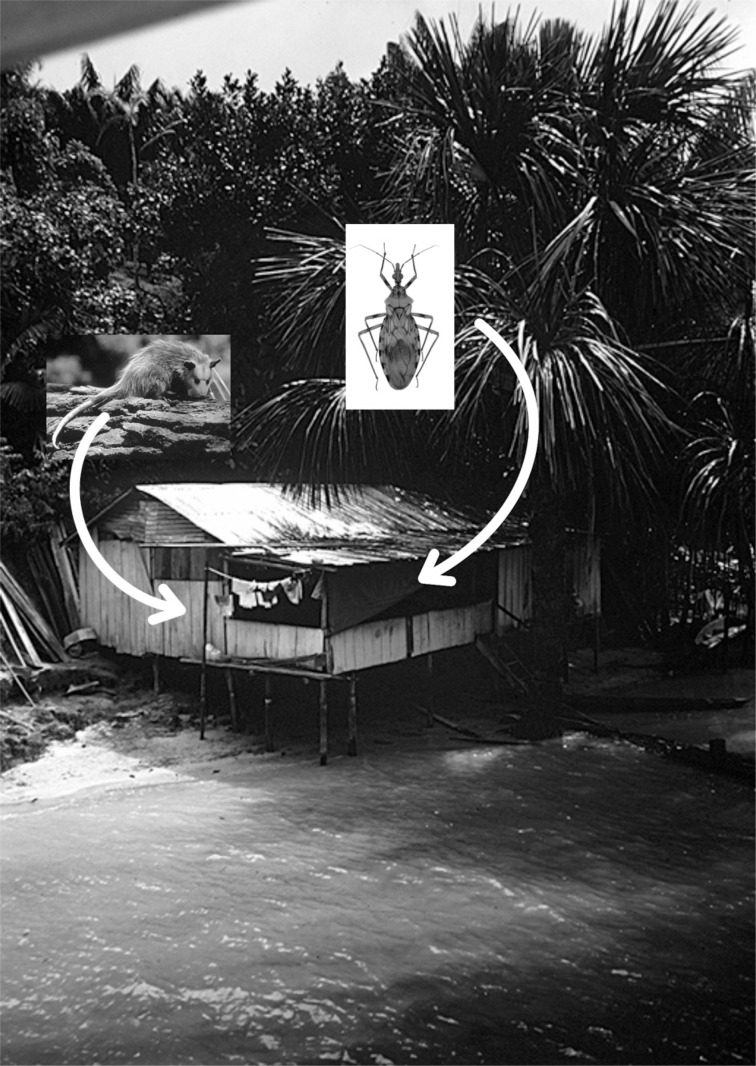
triatomine and marsupial invading human homes.

**Fig. 2:**
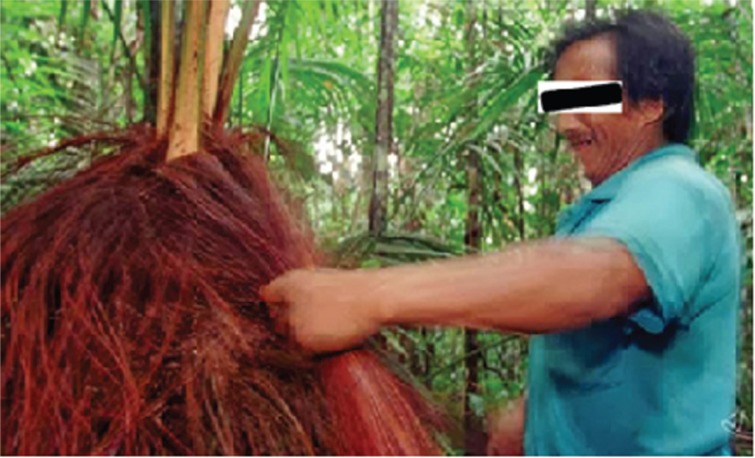
piassaba fibre gatherer.

**Fig. 3:**
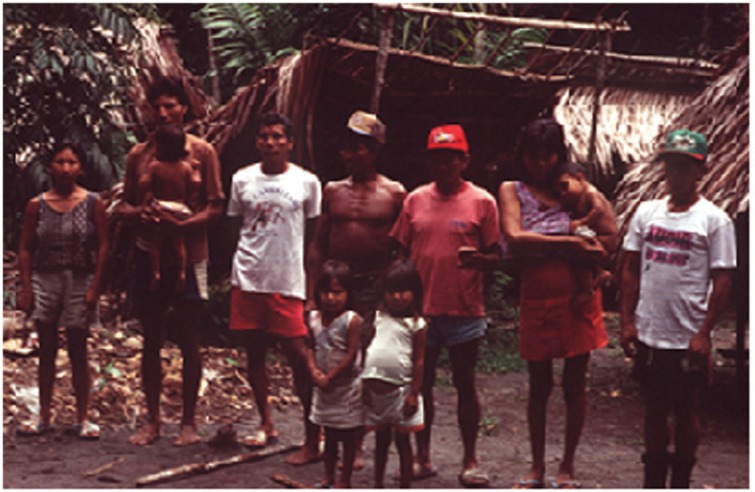
piassaba fibre gatherers and families.

Between when Shaw et al. (1969) described the first four acute autochthonous cases of Chagas disease in Belém, Pará, and 1992, only 38 additional cases of this disease have been described in the Brazilian Amazon Region: 21 in Pará, nine in Amapá, four in Maranhão, three in Amazonas, and one in Acre. Since then, hundreds of cases have been described in acute outbreaks of Chagas disease and serological surveys (Coura et al. 1995a, b, 1999, 2002a, b, Valente et al. 1999, 2009, Pinto et al. 2008, Brum-Soares et al. 2010, and several others).

The risks of Chagas disease have become endemic in the Brazilian Amazon regions and depend on the following factors: (1) the presence of 38 mammal species in six orders: Marsupialia (Didelphiomorphia), Chiroptera, Rodentia, Edentata (Xenarthra), Carnivora, and Primates; (2) the presence of 16 species of wild triatomines, of which 10 are infected with *T. cruzi*, which penetrate homes or come into contact with people when enter forests to hunt or gather plant material (such as piassaba fibres); (3) extensive deforestation involving the displacement of wild animals, which stimulates triatomines to seek blood to feed on, in peridomestic and domestic areas (pigsties, henhouses, and people living in these homes); (4) immigration of people and animals with *T. cruzi* infection from endemic areas to the Amazon Region; and (5) lack of knowledge among people in the Amazon Region, regarding Chagas disease, its transmission mechanisms, and methods of avoiding it.

*Studies of Chagas disease in the Rio Negro microregion, Brazilian Amazon Region* - Over the last 20 years, from 1991 to 2011, we conducted five seroepidemiological and clinical studies of *T*. *cruzi* infection using samples from the population of the Negro microregion in the Brazilian Amazon Region. The surveys included 7286 people living in the main settlement of the municipality of Barcelos, which is in this microregion. The people included in the sample ranged in age from children over the age of one year to elderly people over the age of 60 years. Initially, one blood sample on filter paper was collected per family cluster among the population living in one in four of the inhabited houses (approximately 25%), respectively in 1991, 1993, and 1997. In 2011, blood was collected from the entire population present that permitted collection. Screening tests by immunofluorescence on filter paper was positive in 12.5% of the 710 blood samples collected in 1991, 13.7% of the 658 samples in 1993, and 13.2% of the 886 samples in 1997. However, when we applied indirect immunofluorescence, enzyme-linked immunosorbent assay (ELISA), and western blotting to the serum of the cases that were positive on the filter paper, positivity was confirmed in only 2.8-5% of the 2254 samples collected in 1991, 1993, and 1997. This may be because of an error in interpretation of the technique, in which cases were considered “positive” if immunofluorescent spots were present on the membrane rather than over the entire parasite (Coura et al. 1995a, b, 1999, 2002a, b, 2013). However, for the 4880 blood samples collected from the entire population present in 2011, correction of this error resulted in positivity in only 4.5% of cases (Coura et al. 2013). Similar results may be observed for riverine population of Pará (Valente et al. 1999, 2009, Pinto et al. 2008).

A study of morbidity in which 38 seropositive cases were paired with the same number of age- and sex-matched seronegative cases showed abnormal electrocardiogram results in 36.8% of seropositive cases and 21.5% of seronegative cases, while the echocardiogram results were abnormal in 31.6% of seropositive cases and 18.4% of seronegative cases. Precordial pain and palpitations were observed more frequently in seropositive cases. Radiological examination of the oesophagus showed no differences between seropositive and seronegative cases (Brum-Soares et al. 2010).

*New seroepidemiological survey among piassaba fibre gatherers and members of their families* - A new seroepidemiological survey was conducted in 2015-2016, specifically among piassaba fibre (*Leopoldinia piassaba*) gatherers and members of their families who accompanied them. The locations surveyed were creeks of the Aracá, Curudurí, Demimi, Ererê, and Padauiri rivers, which are tributaries on the left bank of the Negro River, in the municipality of Barcelos (which is 490 km from Manaus, the state capital of Amazonas); Barcelos-Caurés highway; Preto River in Santa Isabel do Rio Negro; and Marié River, on the right bank of the Negro River. Our operational base and field laboratory for this new survey was in the former hospital of the Salesian mission in the main settlement of the municipality of Barcelos. This location formed an extension of our parasitic diseases laboratory at the Oswaldo Cruz Institute (Fiocruz). At this base, fieldwork materials (launches, tents, and other field materials) and bench materials (microscopes, centrifuges, glass cabinets, fridges, freezers and other equipment) were available. Fig. 4 shows the location of the municipalities of Barcelos and Santa Isabel do Rio Negro, and Fig. 5 shows the locations of some of the above mentioned rivers.

**Fig. 4:**
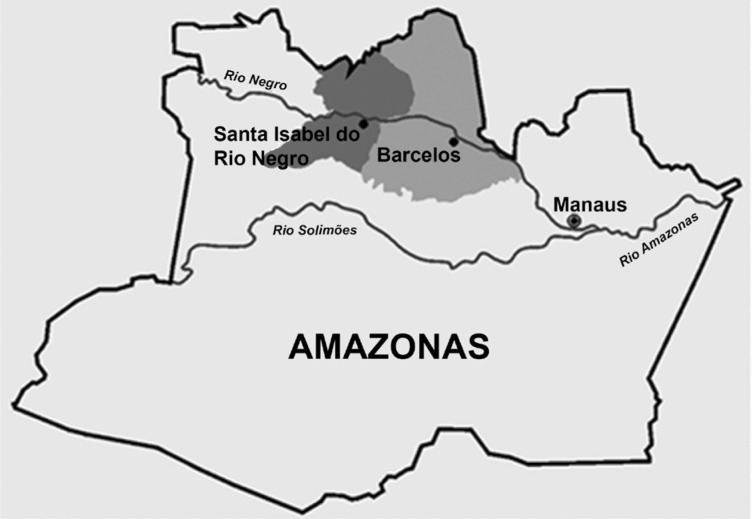
location of municipalities of Barcelos and Santa Isabel do Rio Negro, Amazon.

**Fig. 5:**
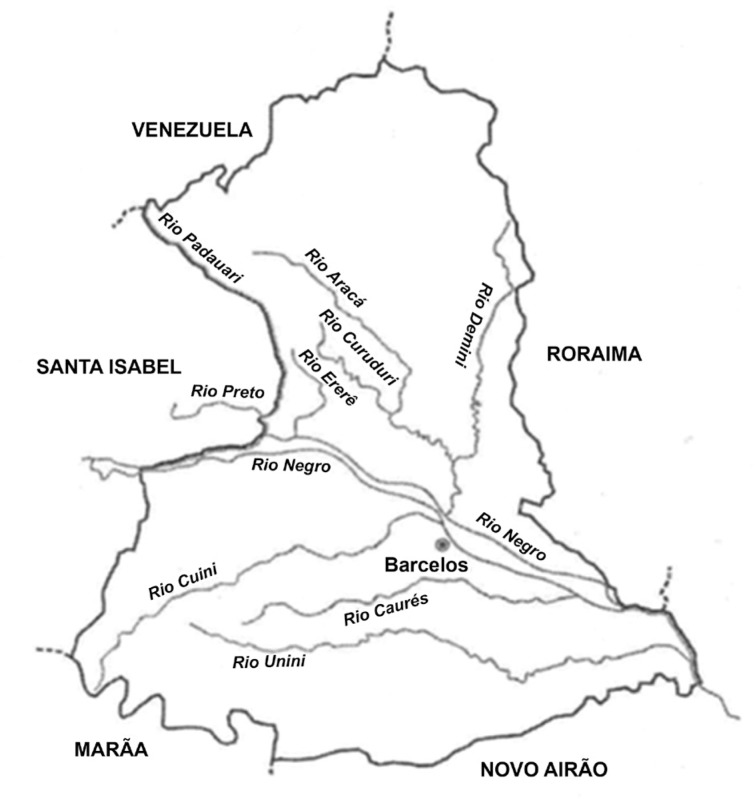
location some rivers of Barcelos e Santa Isabel, Amazonas.

## RESULTS

The questionnaire applied revealed that 100% of the 482 patients (piassaba fibre gatherers and members of their families) recognised the triatomine *Rhodnius brethesi*, which they had observed in the piassaba plantations. Moreover, 81% said that they had seen these triatomines in their shacks and 79% said that they had been bitten between one and ten times by these insects. Only 21% said that they did not know whether they had ever been bitten by these insects. The 25 seropositive patients (Table III) said that they had been bitten more than ten times by these insects.

**TABLE III:** Seropositive patients confirmed for *Trypanosoma cruzi* infection from 482 piassaba-fibre gatherers and families from Rio Negro, Amazon Region (2015-2016)

No.	Sex	Age	Serology	titles	Results
			IIF	ELISA	
1	M	43	320	389	IIF + ELISA positives
2	M	28	640	2133	IIF + ELISA positives
3	M	17	160	605	IIF + ELISA positives
4	M	33	80	2324	IIF + ELISA positives
5	M	53	640	2172	IIF + ELISA positives
6	F	27	640	639	IIF + ELISA positives
7	M	25	40	436	IIF + ELISA positives
8	M	37	40	1966	IIF + ELISA positives
9	M	71	320	1793	IIF + ELISA positives
10	F	56	640	2125	IIF + ELISA positives
11	M	68	80	1167	IIF + ELISA positives
12	M	48	160	927	IIF + ELISA positives
13	F	49	160	1045	IIF + ELISA positives
14	M	80	320	1361	IIF + ELISA positives
15	M	34	80	1265	IIF + ELISA positives
16	M	66	160	1143	IIF + ELISA positives
17	M	51	80	1269	IIF + ELISA positives
18	M	45	80	1389	IIF + ELISA positives
19	M	34	40	339	IIF + ELISA positives
20	F	45	80	1303	IIF + ELISA positives
21	F	59	160	1340	IIF + ELISA positives
22	M	75	80	1130	IIF + ELISA positives
23	F	45	160	1014	IIF + ELISA positives
24	F	82	40	448	IIF + ELISA positives
25	M	39	40	1382	IIF + ELISA positives

Of the 482 piassaba fibre gatherers and members of their families who underwent serological examination, only 273 attended our field base for detailed clinical examination and electrocardiogram. History-taking revealed complaints of palpitations, precordial pain, and dyspnoea upon effort, and clinical examination revealed extrasystoles, tachycardia, and bradycardia. The presence of these conditions was independent of whether the individuals showed positive results in serological examination. Electrocardiographic abnormalities were presented by the 273 patients who underwent this examination, among whom 22% showed conditions that were potentially attributable to Chagas disease. However, only 25 of the 482 cases presented convergent positive serological tests (indirect immunofluorescence + ELISA) that were compatible with infection by *T. cruzi*. Thus, the prevalence of serologically positive cases was 5.2%. Among the 25 serological-positive cases, the average age was 48.4 years (range 17-82 years, median 45 years).

## DISCUSSION

The factors determining the morbidity due to Chagas disease and its evolution in the Negro River micro-region, state of Amazonas, relate directly to the population's intensity of exposure to wild vectors, particularly in piassaba plantations in the region, and types of *T. cruzi* strain (Tc1 and Z3), which still show little adaptation to humans. In studies conducted by Araujo (2000) of 10 *T. cruzi* strains (Tc1 and Z3) in this region (two isolated from humans, two from marsupials, and six from *R. brethesi*), it was shown that these strains had low-virulence characteristics. Analysis of these parameters revealed that the prevalence of *T. cruzi* infection and morbidity of Chagas disease in this region were directly related to the population's intensity of exposure. Exposure resulted from the working conditions of piassaba fire gatherers and possibly from the type of wild strain of *T. cruzi* circulating in the area, as demonstrated by Albajar et al. (2003). These authors reported only two fatal cases of Chagas disease in this area, and Xavier et al. (2006) reported three severe cases of disease leading to death shortly afterwards. The cases described by both authors were of piassaba gatherers subjected to frequent exposure over many years of working in the plantations.

*R. brethesi*, referred to as “piassaba louse” by the local population, has a specific niche habitat in the palm species *L. piassaba*. This species contacts piassaba fibre gatherers in the shacks set up close to the piassaba plantations. Transmission of *T. cruzi* generally occurs through invasion of the shacks by triatomines at night, which search for blood meals from the piassaba fibre gatherers and members of their families when the reservoirs in their natural niche in the palm trees are absent. Occasionally, triatomines attack the population outside of their shacks in search of food.

In the various studies we conducted at many piassaba plantations in the Negro River region, we have collected marsupials, primates, rodents, carnivores, bats, and triatomines (*R. brethesi*) infected with *T. cruzi* to varying degrees. This collection includes 240 animals from five species with different percentages of *T. cruzi* (mean of 22.7%) and 949 triatomines (*R. brethesi*), among which only 19 were infected to give a rate of only 2%. Of the total of 1189 animals and triatomines, 73 were infected with *T. cruzi*, and thus the average was 6.14%. Based on these differences, triatomines use different methods for sucking blood from certain animal species or *T. cruzi* has some difficulty in developing in some blood, leading to a low infection rate of only 2%.

*Comments* - (i) Chagas disease in the Negro River microregion, state of Amazonas, is a wild enzootic disease in which the vectors transmit *T. cruzi* to piassaba fibre gatherers and members of their families, predominantly in their shacks; (ii) the dominant vector for *T. cruzi* in this microregion is *R. brethesi*, which has a specific niche habitat in piassaba palm (*L. piassaba*), from where the adult vectors fly out to the shacks of the piassaba fibre gatherers and members of their families to feed if no animals are present or if it is impossible to suck the animals' blood; (iii) methods for preventing transmission of Chagas disease in this region are very limited. Piassaba gatherers may be able to spray the piassaba palm trees and their shacks (to which the vectors migrate) with insecticide; (iv) use of mosquito nets impregnated with insecticide, for the piassaba gatherers' hammocks, may be recommendable. However, considering the heat in this area, it seems unlikely that a mosquito net would be used properly, i.e. as recommended for combating malaria in some areas; (v) finally, to decrease the rate of this disease, we recommend the following measures, in addition to those stated above: (a) to implement specific courses aimed at elementary school teachers who can provide guidance; (b) to train laboratory technicians and endemic-disease agents in early diagnoses of this disease among individuals in the acute phase and provide immediate treatment; and (c) to provide information to local doctors, nurses, and healthcare agents to enable treatment of acute cases and referral of chronic cases of Chagas disease to regional hospitals.
